# Thermoregulation, incubator humidity, and skincare practices in appropriate for gestational age ultra-low birth weight infants: need for more evidence

**DOI:** 10.1007/s12519-024-00818-x

**Published:** 2024-06-12

**Authors:** Umesh Mishra, Deanne August, Karen Walker, Pranav R. Jani, Mark Tracy

**Affiliations:** 1 Faculty of Medicine and Health, University of Sydney, Sydney, Australia; 2 Department of Neonatology, Westmead Hospital, Westmead, NSW Australia; 3 Grantley Stable Neonatal Unit, Royal Brisbane and Women’s Hospital, Brisbane, Australia; 4 School of Nursing, Midwifery and Social Work, University of Queensland, Brisbane, Australia; 5 Department of Newborn Care, Royal Prince Alfred Hospital, Camperdown, Australia

**Keywords:** Incubator humidity, Neonatal intensive care unit, Skincare, Thermoregulation, Ultra-low birth weight

## Abstract

**Background:**

Although not universal, active care is being offered to infants weighing < 500 g at birth, referred to as ultra-low birth weight (ULBW) infants appropriate for gestational age. These infants have the greatest risk of dying or developing major morbidities. ULBW infants face challenges related to fluid and heat loss as well as skin injury in the initial days of life from extreme anatomical and physiological immaturity of the skin. Although there is an emerging literature on the outcomes of ULBW infants, there is a paucity of evidence to inform practice guidelines for delivering optimal care to this cohort of infants.

**Data sources:**

A comprehensive review of the literature was performed using the PubMed and Embase databases. Searched keywords included “thermoregulation or body temperature regulation”, “incubator humidity”, “skin care”, “infant, extremely low birth weight” and “ultra-low birth weight infants”.

**Results:**

Evidences for thermoregulation, incubator humidity, and skincare practices are available for preterm infants weighing < 1500 g at birth but not specifically for ULBW infants. Studies on thermoregulation, incubator humidity, or skincare practices had a small sample size and did not include a sub-group analysis for ULBW infants. Current practice recommendations in ULBW infants are adopted from research in very and/or extremely low birth weight infants.

**Conclusions:**

This narrative review focuses on challenges in thermoregulation, incubator humidity, and skincare practices in ULBW infants, highlights current research gaps and suggests potential developments for informing practices for improving health outcomes in ULBW infants.

Video abstract (MP4 1,49,115 kb)

**Supplementary Information:**

The online version contains supplementary material available at 10.1007/s12519-024-00818-x.

## Introduction

Advances in maternal and neonatal care have significantly improved survival and health outcomes for extremely low birth weight (ELBW) infants (birth weight < 1000 g). While infants < 1000 g are considered ELBW, there is no international consensus that specifically defines the cohort of infants appropriate for gestational age and weighing < 500 g at birth, usually born at or below 23 weeks’ gestation. We suggest referring to them as “ultra-low birth weight (ULBW) infants”. Although offering care for infants born at this gestation or weight is not universal, there is a growing trend, with some centers now offering active care to ULBW infants and reporting on their survival and health outcomes [1–5]. It is not clear whether the outcome for this small group of extremely small newborns differs from those born at gestational ages 23–25 weeks and birth weights 500 to 750 g. There is also little evidence to guide clinicians and policymakers on evidence-based practices in ULBW infants, including thermoregulation, incubator humidity, and skincare in the first few weeks after birth. These infants have a high risk of dying or developing major morbidities [6]. Therefore, there is an emerging need for an evidence summary for delivering optimal care to this highly vulnerable population. In this narrative review, we aim to present evidence on thermoregulation, incubator humidity and skincare practices in ULBW infants (who are usually born at or below 23 weeks’ gestation), highlight gaps in the literature and provide suggestions for informing future practice for optimizing clinical care for these ULBW infants.

## A global paradigm shift for providing active care to infants born at margins of viability

There has been a global paradigm shift in gestational age for infants born at lower margins for viability. This practice varies across income settings, and infants in many countries are now receiving active care at ≤ 23 weeks’ gestation [2–4, 7]. A recent survey explored the views of parents and clinicians on the practice of considering active care for extremely preterm birth at the margin of viability [8]. Although parents and clinicians have differing views on major risks when considering active care for these infants, both views are important for informing shared decision-making [8]. Information on survival and health outcomes in ULBW infants is emerging from many countries [5, 9, 10]. However, there is little information on their survival in low-income countries. A gap in survival exists for extremely preterm infants (born at < 28 weeks’ gestation) between high-income and low-income countries [11].

## Unique challenges in ultra-low birth weight infants

**Fig. 1:**
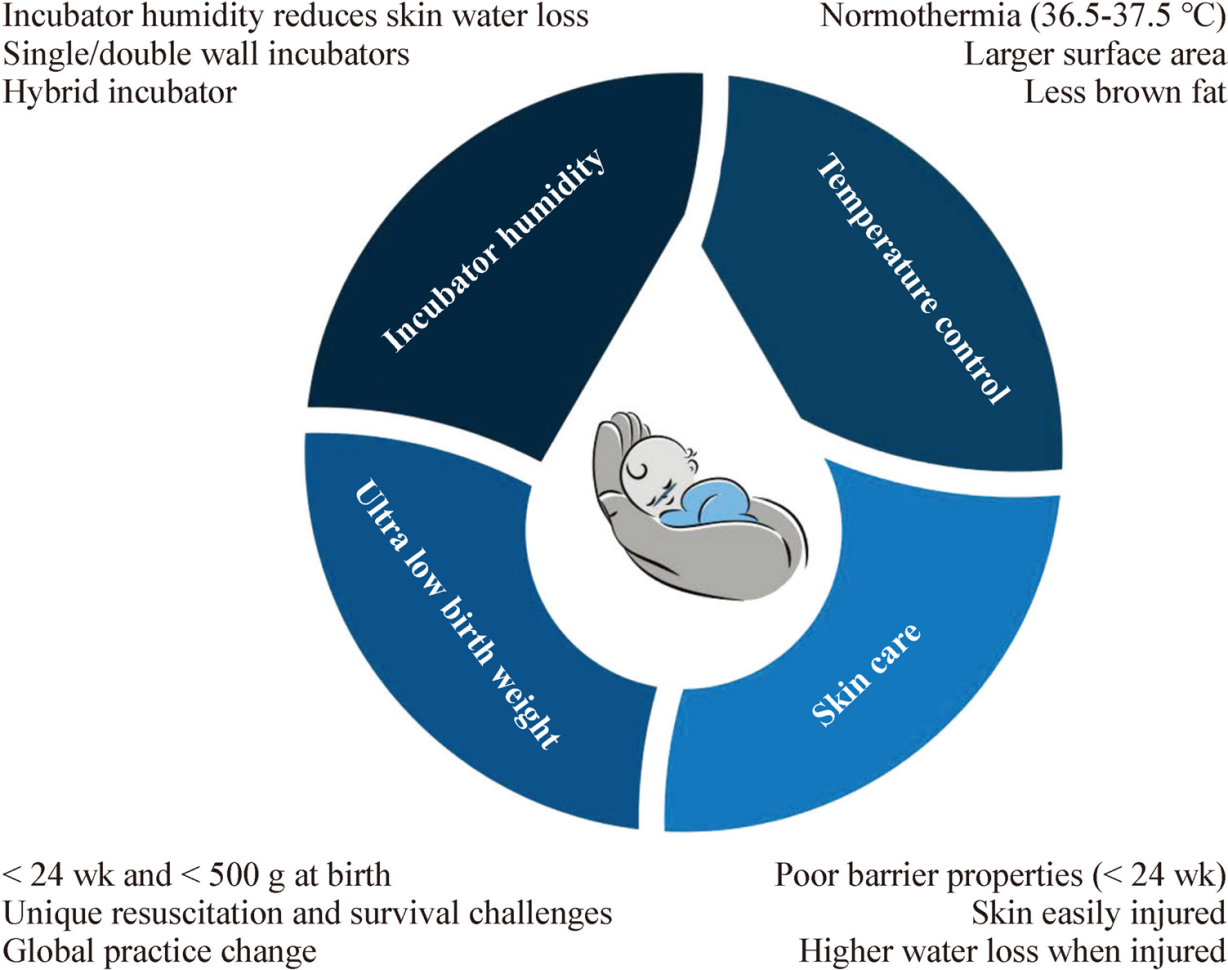
Thermoregulation, incubator humidity and skincare practices in ultra-low birth infants

ULBW infants face challenges related to fluid and heat loss in the initial days of life from underdeveloped skin, which can lead to dehydration and hypothermia. Skin water loss, also known as trans-epidermal water loss (TEWL), is influenced by the maturity of the skin and is inversely related to the infant's gestational age and post-natal age [12]. In this review, we highlight the challenges and gaps in the knowledge of three important and interrelated practices in the care of ULBW infants: maintaining normothermia, maintaining incubator humidity, and maintaining skin conditions (Fig. 1).

## Thermoregulation

### In the delivery room

Thermoregulation, a vital aspect of neonatal care, involves maintaining normothermia (body temperature between 36.5 °C and 37.5 °C) by balancing heat production and heat loss. Maintaining normothermia in term and preterm infants reduces mortality and morbidity [13]. Preterm infants, especially those born extremely premature (< 28 weeks’ gestation), are particularly susceptible to hypothermia due to a greater surface area-to-body mass ratio, lower body fat depots and immature thermoregulatory mechanisms than term-born infants [14, 15]. Maintaining normothermia in ULBW infants involves reducing heat loss from convection, conduction, radiation, and evaporation. In the delivery room (DR), multiple interventions, including the use of polyethylene wrap, thermal mattress, heated humidified gasses, skin-to-skin care, head cover, radiant warmth, and increasing the ambient temperature, are listed in the international resuscitation guidelines for maintaining normothermia during the stabilization of preterm infants ≤ 32 weeks’ gestation [16]. These interventions are often used in combination with each other to maintain normothermia [17]. The quality of evidence judged by the number of studies and small sample size for these interventions included in the Cochrane review at best was moderate to low [18]. As few studies in this Cochrane review included ULBW infants, there is no evidence regarding the effectiveness of the above-listed interventions in ULBW infants [19–21]. Future trials should address this gap in evidence.

#### Heated humidified gasses during delivery room stabilization

Heated and humidified inspired gasses are used during DR stabilization in preterm infants [17]. A meta-analysis of two clinical trials that collectively enrolled 476 preterm infants < 32 weeks’ gestation reported a 36% [95% confidence interval (CI) = 17%–50%] reduction in admission hypothermia and a 39% (95% CI = 10%–58%) reduction in preterm infants < 28 weeks when heated humidified gasses were used compared to cold dry gas for the initial stabilization of preterm infants [22]. The mean or median birth weight for these infants was < 1500 g. However, for infants < 26 weeks included in the above meta-analysis, the difference was not significant, possibly due to the small sample size (*n *= 69). There is a need for further evidence on the short- and long-term benefits of the use of heated humidified inspired gasses in ULBW infants.

#### Optimal ambient temperature of the delivery room

The World Health Organization (WHO) suggests that DRs should be kept at 25 °C to 28 °C (77 °F–82.4 °F) to optimize the maintenance of normothermia for newborn infants [23]. The American Academy of Pediatrics and the American Heart Association have specific recommendations for maintaining the ambient temperature of the DR between 23 °C and 25 °C (74 °F–77 °F) to reduce hypothermia and mortality in preterm infants [24]. A clinical trial reported a lower incidence of hypothermia in premature infants upon admission to the neonatal intensive care unit (NICU) when the DR temperature was 24 °C to 26 °C than when the DR temperature was 20 °C to 23 °C [25]. The ambient temperature in operation theaters is often less than 20 °C, and specific standards for an optimal DR temperature have not been established for ELBW or ULBW infants [26].

### In the neonatal intensive care unit

#### Open versus closed systems for maintaining normothermia

Insensible water loss (IWL) in LBW infants with birth weights < 2500 g is greater when using an overhead radiant warmer (open system) than when using a covered incubator (closed system) [27]. Agren et al. demonstrated similar patterns in IWL using 75% or 50% relative humidity in a small cohort of infants born at 23–27 weeks’ gestation [28]. While an open system allows caregivers easy access to the infant, it may interrupt thermoregulation. A hybrid incubator (a combination of an open and a closed system, which is commercially available) may offer a reasonable compromise to the limitations of the individual system.

#### Manual (air temperature) versus servo (skin servo) control heat modes

An incubator keeps infants warm by providing heat either manually or in servo control mode. In manual mode, the incubator air temperature is maintained at a set temperature, whereas in servo mode, the incubator air temperature is regulated by measuring the infant's temperature using a skin sensor. These non-invasive sensors provide continuous monitoring of body temperature. Both servo and manual modes are used in clinical practice, but limited information exists to suggest whether one heat mode is superior to the other for providing the best possible thermal environment in ULBW infants [17, 29–31]. The main aim of using either heat mode is to achieve a neutral thermal environment (NTE), which maintains a body temperature consistent with minimal oxygen consumption. This NTE is typically achieved between 36.4 °C and 37.28 °C [29]. A randomized trial in 38 well preterm infants < 32 weeks’ gestation at birth demonstrated greater weight gain after 11 days of life when using the manual control mode, suggesting lower body energy consumption [32]. Although the results of this study are encouraging, it was limited by the small sample size of 38 infants, and there was no information on the inclusion of ULBW infants. Future studies are needed on the effects of servo and manual modes on clinical outcomes in ULBW infants.

#### Skin temperature sensor care and sites

Skin temperature sensors offer continuous body temperature measurements compared to intermittent measurements for visualizing trends in temperature over time. Substantial variation in practice exists regarding the site of securing a temperature sensor during DR stabilization and during admission to the NICU for preterm infants [17, 33]. There are no studies comparing the effectiveness of temperature sensors at different sites on thermoregulation in ULBW infants. Although temperature sensors are non-invasive, they may cause skin pressure injury and adhesive-related epidermal stripping [34]. Rotating the site of monitoring devices and using a barrier layer (e.g., hydrocolloid) between the adhesive tape are common practices in NICUs to reduce skin injury [17]. Additional challenges in ULBW infants are maintaining contact between the sensors and the underdeveloped skin when using high incubator humidity. There is a need to improve the understanding of whether adhesive tapes stick well to the infant’s skin when using high incubator humidity (> 90%). When using a skin servo to control heat, care must be taken to ensure that the skin temperature sensor maintains good contact with the infant’s skin.

### Incubator interventions

#### Incubator humidity practices

Since their invention in the 1880s, incubators have been widely used to provide a stable thermal environment and humidity to preterm infants [35]. There is substantial global variation in initial incubator humidity use among extremely preterm infants (ranging from 60% to  > 90%) [17]. Without ambient humidity, the IWL is greater, which may lead to dehydration and hypernatremia (blood sodium level > 145 mmol/L) [35]. Hypernatremia is associated with an increased risk of death, any and/or severe intraventricular hemorrhage (grade III and IV based on Papile’s grading) and childhood disability [36–42]. Despite this association, there is absence of evidence from clinical trials of varying incubator humidity levels addressing these outcomes in ULBW infants. In the absence of national or international guidelines on best incubator humidity practices for ULBW infants, researchers have synthesized existing evidence and developed guidelines for ELBW infant care in the NICU [43]. Evidence from a retrospective study suggested the possible benefits of using 95% initial incubator humidity in infants born at 24 weeks for reducing total fluid intake and electrolyte imbalance in the first few days after birth. However, infants born ≤ 23 weeks in this study continued to have higher IWL, total fluid intake, and electrolyte imbalance despite using 95% humidity [44]. This suggests a need for heightened awareness of the consequences of highly immature skin architecture in ULBW infants. In their clinical trial, Kong et al. examined the effects of 70% compared to 80% incubator humidity on thermal stability in 50 infants born ≤ 28 weeks’ gestation and reported similar thermal stability between the two groups [45]. The rates of skin integrity and colonization of the incubator walls with pathogens were similar for both incubator humidity levels [45]. However, further research is needed to fully understand the relationships among incubator humidity, sepsis, hypernatremia, and intraventricular hemorrhage in this population.

Skin architecture and function are significantly underdeveloped in infants born before 27 weeks’ gestation compared to infants born at term, with only a single layer of epidermis [46]. This anatomical and physiological immaturity is exaggerated in ULBW infants, causing extreme levels of TEWL, dehydration, and hypernatremia. A prospective study (*n* = 22) revealed similar rates of TEWL, IWL, electrolyte imbalance, total fluid intake, and fluid output volumes between infants born at 22–23 weeks’ gestation and those born at 24–25 weeks’ gestation when they were cared for at 95% incubator humidity [47]. Evidence from clinical trials is needed to ascertain the impact of high incubator humidity on TEWL-related outcomes, mortality, morbidities and childhood disability in ULBW infants.

#### Humidity, weaning, and stopping practices of incubators

Little evidence is available to guide clinicians on the best practices for weaning and stopping incubator humidity for ULBW infants. This lack of general international guidelines may have contributed to regional and international variations in practice [17, 48, 49]. Clinicians may consider post-natal age or weight gain before deciding on weaning incubator humidity [48]. Some clinicians wean humidity when temperature and fluid balance stability are achieved and cease incubator humidity at 40% or at 21 days, whichever is earlier [50]. Discontinuation of humidity by two weeks of age or using lower incubator humidity to allow maturation of the stratum corneum has also been suggested [28, 43]. This may contribute to reducing skin colonization by pathogens, thus reducing the risk of developing sepsis.

#### Single versus double-walled incubators

The ambient air and the wall temperature of the patient's environment in the NICU can affect the temperature of a single-wall incubator, thus contributing to radiant heat loss. A double-walled incubator has an inner and outer panel. This reduces the exposure of the inner panel to ambient air and the wall temperature. Compared with a single-wall incubator, a double-walled incubator offers enhanced insulation and creates a better thermo-neutral environment for ELBW infants [51–53]. Although its use reduces heat loss and oxygen consumption, further evidence is needed on its benefits on long-term outcomes such as mortality or length of hospital stay [54].

#### Hybrid versus conventional incubators

Hybrid incubators offer versatile functioning as both an open system (radiant warmer) and a closed system (incubator for providing humidity) [55]. The choice between accessing the infant through open portholes and by raising the canopy depends on various factors, including caregiver preference, medical condition, family presence, and urgency of care. Lifting the canopy of the incubator may allow clinicians better access to infants for performing procedures such as the insertion of umbilical catheters. For ELBW infants in the first week of life, better thermoregulation was achieved by minimizing opening the incubator side panels and using portholes instead of lifting the canopy. Additional functions may result in better thermal stability when accessing infants by opening the portholes [54]. This may allow swift adaptation to changing clinical needs, offering an optimal thermal environment. A number of leading companies are now manufacturing hybrid incubators [55]. While there are limited studies that included ULBW infants, evidence suggests that using humidified hybrid incubators in comparison to non-humidified incubators may enhance care for ELBW infants by lowering total fluid intake, improving electrolyte balance, and enhancing growth velocity without compromising thermoregulation [55]. The incidence of severe bronchopulmonary dysplasia and the duration of assisted ventilation were lower in the humidified hybrid incubator group than in the non-humidified incubator group, possibly because of the lower total fluid intake in the humidified hybrid incubator group [56]. Optimizing fluid management (that is, lowering the total fluid intake) in addition to using these devices holds promise in potentially reducing severity of bronchopulmonary dysplasia.

#### Rainout from high humidity use

Additional condensation (water) on the inner walls of the incubator may occur when using high incubator humidity (> 90%), known as rainout. This can be mitigated by wiping the inside of the incubator with a dry cloth or using double-walled incubators, covering the sides and the canopy of the incubator with a dedicated cover and maintaining a higher ambient temperature in the infant’s room. The inability to adequately visualize the infant by their caregivers and families may be a barrier to using high incubator humidity in the first few days after birth. Colonization of incubators with pathogens and concerns about systemic infections with high incubator humidity have been reported; however, there is a gap in evidence to support these concerns [51, 52].

#### Effects of skin-to-skin care and emollient/oil application on trans-epidermal water loss

In preterm infants, the WHO strongly recommends skin-to-skin contact by kangaroo mothers and consideration of topical sunflower or coconut oil; both may impact the delivery of incubator humidity and TEWL, as applying coconut oil to VLBW infants reduces TEWL [57]. Although there are some emerging data, the effects of combined skin-to-skin care and coconut oil use on TEWL in ULBW infants are unknown [58].

### Skincare practices

Skin conditions are intricately linked with thermal regulation and caring in a humidified environment [59]. ELBW infants, skin care practices, and TEWL are closely related. The immaturity of the barrier function of the skin makes it more permeable and susceptible to TEWL. A low humidity environment can exacerbate TEWL, leading to increased skin dryness and potential complications such as skin breakdown and infection [59]. Skin care practices for these infants focus on maintaining skin hydration and integrity and reducing TEWL, as presented in the following sections.

#### Structure and function of the skin in newborn infants

The skin plays a vital role in barrier and immunological responses, thermoregulation, hydration, sensory perception (pain and touch), vitamin production and synthesis, and controlling the absorption of substances [60]. The barrier properties of the epidermis are primarily dependent on the integrity of the stratum corneum. Between 22 and 24 weeks’ gestation, only a single layer of basal cells called the periderm protects the developing epidermis. This layer disintegrates and contributes to the developing vernix, which covers and protects the developing epidermis during the remaining in utero period [46]. However, by the 30th week of gestation, fetal skin structures are more structurally similar to the skin of full-term infants but still have several functional limitations. Despite structural maturation with increasing gestation, skin barrier function still varies across gestational ages, ranging from almost entirely deficient to close to competent, which has significant clinical implications [60]. First, the rate of water loss through the skin is inversely related to gestational age at birth. Second, the absence of barrier function poses substantial risks, such as increased vulnerability to percutaneous absorption of hazardous substances, microbial invasion and colonization, further barrier disruption, and pain or discomfort. In response to any barrier disruption, such as skin injury or wounds, an increase in trans-epidermal water flux serves as a key signal for epidermal proliferation and subsequent keratinization [60]. In extremely preterm infants, the necessary epidermal hyper-proliferative process accelerates between 4 and 10 weeks after birth, which can result in drying, scaling and cracking, resulting in generalized “skin breakdown”. Although the structural adaptation of skin in preterm infants has evolved, the risk for mechanical injury (e.g., pressure, friction, shear, and stripping) remains high due to the slow nature of connective structures (rete ridges and fibers) between dermis and epidermis [61, 62]. In addition to barrier function, the skin and its microbiome play major roles in immune function. Advances in molecular biology have highlighted the composition of the skin microbiome and its relationship with other organs [60]. However, questions about the impact of preterm delivery on the skin microbiome and environmental influences on skin maturation remain unanswered.

#### Skin injuries

Diaper dermatitis and medical adhesive-related skin injuries are common skin injuries in extremely preterm infants [63]. Additionally, regional and resource setting-based variations exist for skin injuries and skincare practices for these infants. In addition to barrier function, the skin and its microbiome play major roles in immune function. Advances in molecular biology have highlighted the composition of the skin microbiome and its relationship with other organs [60]. There is a growing body of evidence on the risk factors contributing to skin injuries and strategies, often a combination of practices referred to as “practice bundles”, to reduce these injuries in ELBW infants [62]. There is a need to report this information for ULBW infants.

#### Emollients and skin antisepsis

Topical emollients and skin barrier preparations (dimethicone or other) have shown promise for reducing nosocomial sepsis and potentially improving the survival of extremely preterm infants; however, their benefits for ULBW infants need further research [64, 65]. Chlorhexidine solutions are effective skin cleansing agents, but they can cause chemical burns in preterm infants when used at relatively high concentrations [66, 67]. There is evidence supporting the use of 2% chlorhexidine gluconate over 0.5% chlorhexidine gluconate in 70% alcohol for skin disinfection before vascular access insertion as it is linked to fewer skin injuries and comparable rates of central-line-associated bloodstream infections [68, 69]. Therefore, the volume and the duration of antiseptic skin cleansing solutions should be approached judiciously, as regular use can delay skin healing, and their use should be focused on procedures and infection prevention [70].

#### Current evidence

Despite growing evidence of injury and risk for ELBW infants, there is limited evidence available for injury prevention and even less evidence for injury management in ULBW infants. Recommendations for skin injury prevention in extremely preterm infants include minimizing the use of adhesive tapes, opting for silicone adhesives, using correct adhesive application and removal techniques, rotating devices and offloading devices when possible [63, 70, 71]. Polyurethane adhesives (e.g., transparent films) allow easy inspection of the underlying skin. These are non-occlusive and are used to secure medical devices but are often sized to suit the pediatric population; hence, they must be trimmed or adapted for ULBW infants. When removing adhesives, it is essential to adopt a gentle approach by pulling tapes horizontally after wetting the adhesive [63]. While there is some evidence to support adhesive remover wipes [mineral or petrolatum oil(s) or silicone], it is important to note that their safety in the first two weeks after birth has not been fully evaluated, and they can make readhesion of tapes to the skin difficult [63, 71]. Therefore, it is advisable to use them with caution during this period. To avoid pressure trauma from medical tubes such as endotracheal and nasogastric tubes, placing hydrocolloid-based dressings underneath acrylate tapes is recommended. Alternatively, polyurethane adhesives found in transparent dressings are non-occlusive (permeable) and allow better access for inspection of the underlying skin, securement effectiveness and injury equal to those of acrylate adhesives (*n* = 57) in a mature neonatal population [72]. As clinicians provide care to ULBW infants, it is important to consider these recommendations when managing injuries related to adhesive use, even in the absence of extensive evidence. Optimizing a conservative approach for managing skin injury involves a combination of measures, including gentle cleansing, keeping the skin dry and using a gentle barrier such as silicone [73].

## Practical implications and future directions

The shorter the gestation period and the greater the weight of a preterm infant, the more complicated their care is due to a higher level of biological immaturity. Stabilizing the infant’s body temperature quickly and minimizing adverse effects on their health, particularly those related to skin care, could be performed in a two-stage personalized process. The first is called control aimed at taking the preterm infant from an undesirable energetic state to the desirable state of lowest metabolic waste, and the second is called stabilization aimed at maintaining it in the desirable state, preventing it from returning to the initial state. The complexity of the energy balance between the production of metabolic heat due to bioenergetic processes and the loss of body heat due to interactions with the surrounding environment through conduction, evaporation, convection, and radiation. Therefore, due to this complexity, personalized treatment is required for each ULBW infant. Table 1 presents a summary of the current evidence on thermoregulation, incubator humidity and skincare practices in ULBW infants. This evidence to inform practice comes mainly from studies in LBW and VLBW infants. There is a growing need to generate evidence, reduce practice variation by standardizing quality improvement initiatives and undertaking collaborative learning to inform current and future practices on thermoregulation, incubator humidity and skincare and improve health outcomes in ULBW infants [13, 17]. This issue has been highlighted in a recent systematic review on proactive treatment at 22 weeks’ gestation [1]. Future research may consider examining whether ULBW infants are simply smaller versions of ELBW infants or whether there are specific factors that need detailed in-depth exploration to optimize technology for delivering better care to ULBW infants. Does the immaturity of the circulatory system in ULBW infants influence heat transfer in biological tissues? Further research may address this question by incorporating models such as the one from Fraguela et al. for maintaining thermal stability in a premature infant cared for in an incubator [74].

**Table 1:** Summary of thermoregulation, incubator humidity and skincare practices

Practices	Summary of key practices in ULBW infants	Outcomes/issues
Thermoregulation	Ensure measures for minimizing heat loss are instituted in the DR and in the NICU	Reduce hypothermia
	Keep the DR warm, at least 25 °C	Reduce hypothermia
	Heated and humidified inspired gasses	Reduce hypothermia
	Thermoregulation can be maintained in either a servo or a manual mode	Achieve NTE Maintaining body temperature consistent with minimal oxygen consumption
	Substantial variation in practice exists regarding the site of securing a temperature sensor	Need further research
Incubator humidity	Ambient incubator humidity reduces IWL, dehydration and hypernatremia	Effects on health outcomes needs further research
	Best practice for weaning and stopping incubator humidity remains unknown	Possible benefit for skin maturation
	Avoid lifting the canopy of the incubator to optimize humidity delivery	Preventing excessive TEWL and maintaining skin integrity
	Ensure rainout mitigation measures are used when using high incubator humidity	Improves patient visibility and clinical monitoring
	The effects of performing skin-to-skin care and of using coconut oil on TEWL are not known	May reduce TEWL
	Substantial variation in practice exists regarding the initial incubator humidity level	Need more research
Skincare	NICU is a high-risk environment for skin injuries to occur and skin injuries are common Evidence for injury prevention and injury management is limited	Reduction in skin injuries and infection Ensure strategies for reducing skin injury are used Promote the use of standardized skin assessment tool

*ULBW* ultra-low birth weight, *DR* delivery room, *NICU* neonatal intensive care unit, *IWL* insensible water loss, *TEWL* trans-epidermal water loss, *NTE* neutral thermal environment

## Conclusions

Recent research regarding the effects of the physical environment on preterm infants highlights the need for an individualized “microenvironment” tailored to meet the infant’s gestational age and medical condition [75–77]. Collaboration between industry, healthcare providers, and consumers is vital for designing incubators that are tailored to meet the needs of all stakeholders. Features for future incubators may include enhanced monitoring and safety capability, ergonomic design, use of sustainable and environmentally friendly materials, integration of smart technology, advanced infection control measures, ability to obtain radiographs without opening side panels, and audio–video capacity allowing remote access to caregivers.

## Data Availability

All data generated or analyzed during this study are included in this published article.
